# Pressing Issues for Oral Health Care Access and Quality Improvement During Pregnancy in Portugal—A Qualitative Study

**DOI:** 10.1111/jphd.70014

**Published:** 2025-10-30

**Authors:** Leonor Frey‐Furtado, Paulo Melo, Stefan Listl, Maria Lurdes Pereira

**Affiliations:** ^1^ EPIUnit of Instituto de Saúde Pública da Universidade do Porto Porto Portugal; ^2^ Laboratory for Integrative and Translational Research in Population Health (ITR) of Instituto de Saúde Pública da Universidade do Porto Porto Portugal; ^3^ Faculdade de Medicina Dentária Universidade do Porto Porto Portugal; ^4^ Department of Dentistry—Quality and Safety of Oral Healthcare Radboud University Medical Center—Radboud Institute for Health Sciences (RIHS) Nijmegen Gelderland the Netherlands; ^5^ Section for Oral Health Heidelberg Institute of Global Health, Heidelberg University Hospital Heidelberg Baden‐Württemberg Germany

## Abstract

**Objective:**

To identify and prioritize pressing issues related to access and quality of oral healthcare during preconception, pregnancy, and postpartum in Portugal.

**Methods:**

Ethical approval (number 8/2024) and data protection clearance (R‐12/2024) were granted. Twelve stakeholders, including healthcare professionals, policymakers, representatives of nongovernmental organizations, and recent mothers, were recruited through snowball sampling. The Nominal Group Technique structured a four‐step process: (1) online discussions at policy, community, and practice levels; (2) initial voting to screen pressing issues; (3) plenary discussion; and (4) final voting.

**Results:**

At the policy level, the highest‐ranked pressing issues were the lack of a mandatory oral health diagnostic appointment for early detection, limited coverage of the *Cheque‐Dentista* program, which excludes the preconception period, and the absence of oral health education in childbirth and parenting courses. At the community level, pressing issues included the absence of oral health in family planning consultations, insufficient oral health training for midwives and nurses, and weak direct engagement with pregnant women through targeted promotion strategies. At the practice level, the most pressing issues were the exclusion of dentists from maternal healthcare teams, the lack of structured oral health appointments in primary healthcare centers, and the omission of oral health modules from childbirth preparation programs.

**Conclusion:**

The Nominal Group Technique enabled consensus‐building and prioritization of concrete pressing issues, producing actionable strategies to strengthen maternal oral healthcare in Portugal. Literacy, accessibility, and collaboration have emerged as foundational elements for integrating oral health into maternal care pathways.

## 1 Introduction

Oral health is essential to overall well‐being and quality of life, yet untreated oral diseases remain a global public health problem, affecting billions of people despite their preventable nature [1]. Pregnancy is characterized by natural physiological changes that can affect women's physical structure, hormonal levels, metabolism, and immune response, which heightens the risk of having oral health problems [2]. However, a significant proportion of pregnant women face barriers to accessing oral healthcare [3]. These barriers encompass limited health literacy, inadequate accessibility, and challenges related to healthcare quality dimensions, including safety, effectiveness, person‐centeredness, timeliness, efficiency, and equity [4].

Oral diseases reduce an individual's quality of life and significantly affect societal productivity and economic stability [5]. Globally, the economic burden of poor oral health in 2019 reached $710 billion, including $387 billion in treatment costs and $323 billion in lost productivity [6]. The shared risk factors with other noncommunicable diseases (NCDs), such as sugar consumption, compound health disparities and exacerbate healthcare system pressures [7]. Pregnant women, as a vulnerable population, face increased disparities, requiring specific interventions to improve access to and quality of oral healthcare. Portugal's *Cheque‐Dentista* Program, launched in 2008, provides dental vouchers to specific patient groups, including pregnant women, enabling them to access oral healthcare provided by private dentists who adhere to the program [8]. Despite its potential, the program's utilization rate remains below 70%, highlighting the need for improved outreach and engagement [9]. Moreover, unlike England's NHS system, which guarantees free dental care during pregnancy and postpartum [10], Portuguese policy relies on partial coverage. Importantly, there is a lack of recent national data on oral health, since the last national oral health survey was carried out in 2013/2014. This data gap limits both research and informed policymaking. Existing Portuguese data and studies have mainly focused on documenting utilization rates of the *Cheque‐Dentista* Program or knowledge and attitude patterns among pregnant women [11, 12, 13, 14]. However, to our knowledge, no study has involved multiple stakeholders—including mothers, healthcare professionals, and policymakers—in reaching a consensus on priorities for improving maternal oral healthcare.

Literature highlights the importance of co‐creating health solutions with diverse stakeholders to enhance the efficiency, acceptability, and adaptability of interventions [15]. Collaborative efforts involving citizens, healthcare professionals, and policymakers are essential to comprehensively address access barriers and delivery challenges. This study aims to collaboratively identify and prioritize pressing issues related to access and the delivery of oral healthcare during preconception, pregnancy, and postpartum periods in Portugal. By addressing these challenges across practice, community, and policy levels, we aim to develop targeted, evidence‐based solutions that may improve overall and oral health outcomes.

## 2 Materials and Methods

Data protection (R‐12/2024) and ethical permission for this research (no. 8/2024) were obtained from the Ethics Committee for Health and the Data Protection Department. All participants were provided with a detailed summary of the study's objectives and data protection measures, and informed permission was obtained from each subject prior to their participation.

This study employed the Nominal Group Technique (NGT), a structured four‐stage process consensus‐building method recognized for its effectiveness in exploring issues, generating ideas, and achieving consensus [16, 17, 18]. NGT was selected over alternatives (e.g., Delphi, focus groups) because this approach includes both individual and collaborative efforts to ensure that all participants are given equal opportunities for engagement and contribution [16, 17, 18].

To identify and prioritize pressing issues related to access and delivery of oral healthcare during preconception, pregnancy, and postpartum periods in Portugal, we recruited 12 participants from different backgrounds through a snowball technique to capture diverse expertise: a pediatric dentist, a dentist working in a primary care center, a general practitioner, a public health researcher, recent mothers, health policymakers, and workers (coordinator and clinic director) at a clinic integrated into a nongovernmental organization that promotes oral health among populations in situations of socioeconomic vulnerability. Due to the difficulty of finding pregnant women for this study, we only included recent mothers who had given birth within the last 2 years. This sample may not represent the national opinion, as perspectives on oral healthcare may differ across Portugal. However, the use of snowball sampling enabled us to recruit participants from diverse professional and personal backgrounds, ensuring the heterogeneity of viewpoints and providing a solid exploratory basis for identifying priority areas for future research and policy discussion.

A systematic review of global barriers to oral healthcare during pregnancy was conducted to compile an initial list of pressing issues [3]. This list informed Stage 1 of the NGT, grounding discussions in existing literature while allowing participants to propose additional issues. The research team internally validated and adapted the list to the Portuguese context before distributing it to participants. The pressing issues were then categorized into three analytical levels: the policy level, referring to measures requiring governmental or organizational action; the community level, targeting population groups or local contexts; and the practice level, addressing actions at the provider–patient interface.

### 2.1 Stage 1: Online Group Discussion (November 7, 2024)

Before the meeting, participants received the preliminary list of pressing issues and were instructed to prepare brief statements (based on the silent idea generation process). At the start of the online session, study procedures were explained, and participants were allocated to thematic groups based on their professional background and lived experience to ensure relevance within each domain, which we believed facilitated collaborative enhancement of classifications through stakeholder perspectives:

- *Practice‐level group*: One recent mother, one pediatric dentist, and one primary care dentist.
- *Community‐level group*: Two nongovernmental organization professionals (coordinator and clinic director) working with socioeconomically vulnerable populations, two recent mothers, and a general practitioner.
- *Policy‐level group*: One recent mother, one health policymaker, a dentist, and a public health researcher.

Each group met in a separate online room facilitated by a moderator. Using a structured round‐robin approach, participants sequentially presented their ideas in short statements, which the moderator documented with minimal paraphrasing. The Round–Robin discussion process within each group enabled participants to propose modifications or reclassifications based on their expertise and understanding of the Portuguese oral healthcare context.

### 2.2 Stage 2: Initial Voting

To refine the lists, we used an online voting process based on a licensed Google Form from the University of Porto. Each issue was evaluated using the following options: *Yes*, *No*, *Abstain*, or *Abstain due to conflict of interest*. Pressing issues receiving less than 75% “Yes” votes were excluded. The updated lists for each theme were emailed to all participants.

### 2.3 Stage 3: Plenary Online Meeting (November 28, 2024)

The three group moderators presented their thematic lists to all participants in the 30‐min plenary meeting. This session provided an opportunity for open discussion, during which participants could offer additional insights or propose modifications of the pressing issues lists. Moderators wrote all new contributions and suggestions in real time.

### 2.4 Stage 4: Final Voting and Prioritization

In an online voting process, participants were asked to rate the importance of each remaining pressing issue on a Likert scale ranging from 1 (*least important*) to 5 (*most important*). Pressing issues with an average score of two or below were excluded from the final list. Statistical analysis assessed the distribution of scores, ensured internal consistency, and enabled the generation of a prioritized list of pressing issues, ranked by their average scores.

The pressing issues for oral care quality improvement were ranked by calculating mean scores for each item across levels in Microsoft Excel. Within each level, a mean of means was computed to establish a cut‐off point. Final prioritization was determined by retaining only those issues exceeding this cut‐off. This threshold methodology, developed in previous research [19], addresses a limitation of NGT: the lack of systematic criteria for constructing final priority lists. Traditional approaches often generate unwieldy, overly comprehensive lists. Our method instead derives level‐specific thresholds directly from stakeholder rating distributions, ensuring that only pressing issues rated above the average importance within each domain are prioritized.

## 3 Results

**FIGURE 1:**
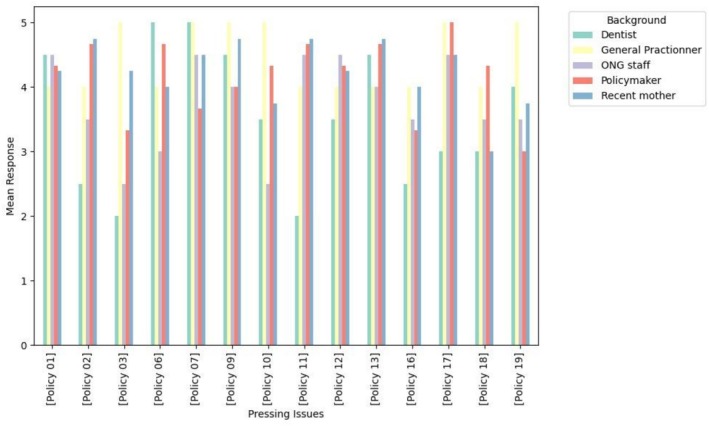
Policy‐level pressing issues scores. Bars represent the stakeholder rating of importance (Likert scale 1–5), with higher scores indicating stronger prioritization. [Color figure can be viewed at wileyonlinelibrary.com]

**FIGURE 2:**
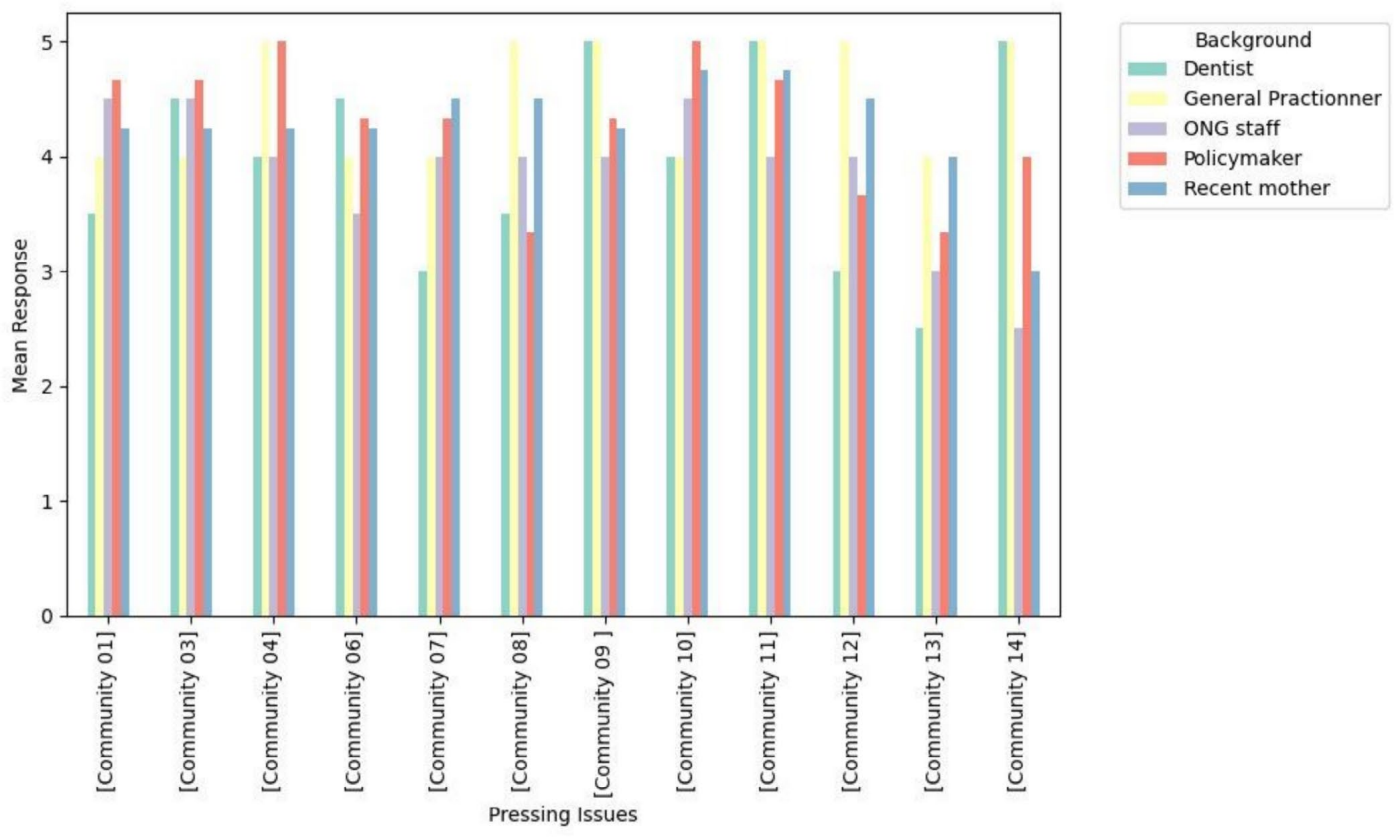
Community‐level pressing issues scores. Bars represent the average stakeholder rating of importance (Likert scale 1–5), with higher scores indicating stronger prioritization. [Color figure can be viewed at wileyonlinelibrary.com]

**FIGURE 3:**
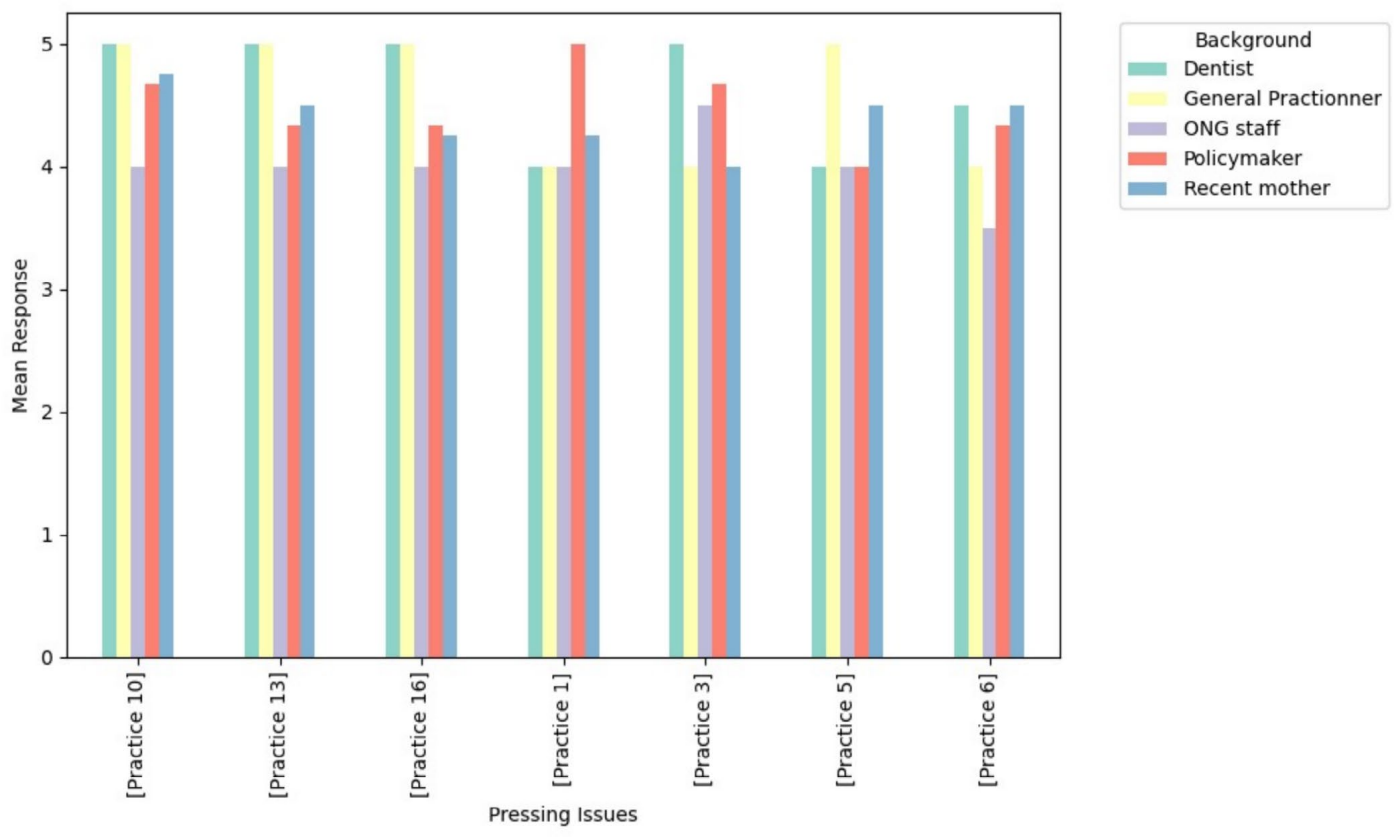
Practice‐level pressing issues scores. Bars represent the average stakeholder rating of importance (Likert scale 1–5), with higher scores indicating stronger prioritization. [Color figure can be viewed at wileyonlinelibrary.com]

The present study outlines the results of a prioritization process that involved 12 stakeholders. The prioritization process revealed consensus on the most urgent oral health issues across policy (Table 1), community (Table 2), and practice levels (Table 3), while also highlighting distinct stakeholder perspectives (Figures 1, 2, 3).

**TABLE 1:** Distribution of scores linked to pressing policy issues.

ID	Policy pressing issues (prioritized)	Mean (min—max)
P13	Include an oral health diagnostic appointment in primary care provided to pregnant women, to detect and treat oral health problems at an early stage.^a^	4.5 (4–5)
P17	Increase the number of vouchers of *Cheque Dentista* Program and expand their use to the pre‐conception period, guaranteeing preventive care before pregnancy.^a^	4.4 (3–5)
P09	Include a compulsory module on oral health in childbirth and parenting preparation courses.^a^	4.4 (3–5)
P07	Integrate oral health into general health.^a^	4.4 (3–5)
P01	Increase access to dental care for pregnant women.^a^	4.3 (3–5)
P12	Match the number of dental vouchers to the individual needs of pregnant women.^a^	4.2 (3–5)
P11	Allow obstetricians to issue vouchers of *Cheque Dentista* Program.^a^	4.2 (1–5)
P06	Offer specific training (undergraduate and postgraduate) in oral health for other health professionals who treat pregnant women.^a^	4.2 (2–5)
P02	Intensify the dissemination of programs such as the *Cheque Dentista* Prgramme.^a^	4.1 (2–5)
P19	Ensure preventive strategies for women's health, including oral health (in a global sense, not just focused on pregnant women).	3.6 (3–5)
P18	Ensure the implementation of effective oral health communication strategies at national level (e.g., using influencers…).	3.5 (1–5)
P16	Facilitate access to oral hygiene products for pregnant women through partnerships with pharmacies.	3.5 (1–5)
P10	Make it easier for pregnant women to use health subsystems, with incentives for dentists and timely payments.	3.5 (2–5)
P03	Ensure oral health insurance coverage with a focus on preventive treatment for pregnant women, guaranteeing accessibility and effectiveness.	3.4 (1–5)

^a^ Prioritized pressing issue above cut‐off.

**TABLE 2:** Distribution of scores linked to pressing community issues.

ID	Community pressing issues (prioritized)	Mean (min—max)
C11	Introduce the importance of oral health in family planning consultations (preconception).^a^	4.7 (4–5)
C10	Reinforce oral health training for health professionals. Promoting more awareness—ensuring that the general practitioner, as the main link with the pregnant woman addresses this issue and issues vouchers of *Cheque Dentista* Program.^a^	4.6 (4–5)
C09	Convene pregnant women directly and independently to address oral health promotion and other important topics (there isn't time for everything at the first appointment with general practitioner).^a^	4.4 (3–5)
C12	Advancement of the *Cheque Dentista* Program, allowing for the issuance of an increased number of vouchers based on user requirements. Including the delivery by other healthcare professionals.	4.4 (3–5)
C01	Greater accessibility—ensuring that pregnant women have easier access to dental services.^a^	4.3 (3–5)
C03	Increase oral health literacy among pregnant women.^a^	4.2 (4–5)
C04	Promote appropriate oral health practices during pregnancy.^a^	4.2 (3–5)
C06	Use more media to disseminate information about the type of oral health support available.	4.1 (3–5)
C07	Make information about access to primary oral health care clear. Consistent and organized (e.g., program available, where to find dentists who accept vouchers of *Cheque Dentista* Program, how to access health centers).	4.1 (3–5)
C08	Postpartum follow‐up dental appointments (e.g., with Cheque Dentista).	4.0 (3–5)
C14	Create a specific consultation for pregnant women at the dentistry universities (direct approach by appointment).	3.7 (2–5)
C13	Offer a kit with basic oral hygiene products in health centers or dental appointments.	3.4 (1–5)

^a^ Prioritized pressing issue above cut‐off.

**TABLE 3:** Distribution of scores linked to pressing practice issues.

ID	Practice pressing issues (prioritized)	Mean (min–max)
Pr10	Incorporate a module on oral health within childbirth preparation training.^a^	4.7 (4–5)
Pr13	Include dentists in pregnancy support teams.^a^	4.5 (4–5)
Pr16	Establish oral health consultations for pregnant women in health centers, involving multidisciplinary teams that prioritize prompt diagnosis and efficient referrals to the *Cheque Dentista* Program or dental schools.^a^	4.4 (3–5)
Pr03	Provide oral health training for general health professionals who assist pregnant women.^a^	4.4 (3–5)
Pr06	Adhere to national guidelines regarding dental care during pregnancy.	4.3 (3–5)
Pr05	Implement interdisciplinary care model for pregnant women.	4.3 (3–5)
Pr01	Ensure sufficient accessibility through comprehensive coverage of dental care for pregnant women, which is currently inadequate.	4.3 (2–5)

^a^ Prioritized pressing issue above cut‐off.

At the policy level, stakeholders prioritized the integration of oral health into general health services (P07), mandatory oral health modules in childbirth and parenting courses (P09), and the inclusion of a diagnostic appointment in primary care (P13). While all three were highly rated, recent mothers consistently rated P13 highest, reflecting the perceived value of immediate, accessible diagnostic services. In contrast, general practitioners and policymakers emphasized P07 and P09, pointing to systemic reforms and structured health education as policy priorities.

At the community level, the most pressing issues included introducing oral health during family planning consultations (C11), directly engaging pregnant women for oral health promotion (C09), and enhancing training for general practitioners (C10). Dentists and GPs prioritized C11, emphasizing prevention in the preconception phase, while NGO representatives highlighted C09. Policymakers assigned greater importance to C10, reflecting concern with system‐wide professional capacity.

At the practice level, the most voted pressing issues were integrating dentists into pregnancy support teams (Pr13) and establishing structured oral health consultations with referral capacity in health centers (Pr16). These were consistently prioritized by clinicians, who stressed their feasibility and impact on care delivery. Conversely, mothers placed greater emphasis on oral health modules embedded in childbirth preparation programs (Pr10).

## 4 Discussion

**FIGURE 4:**
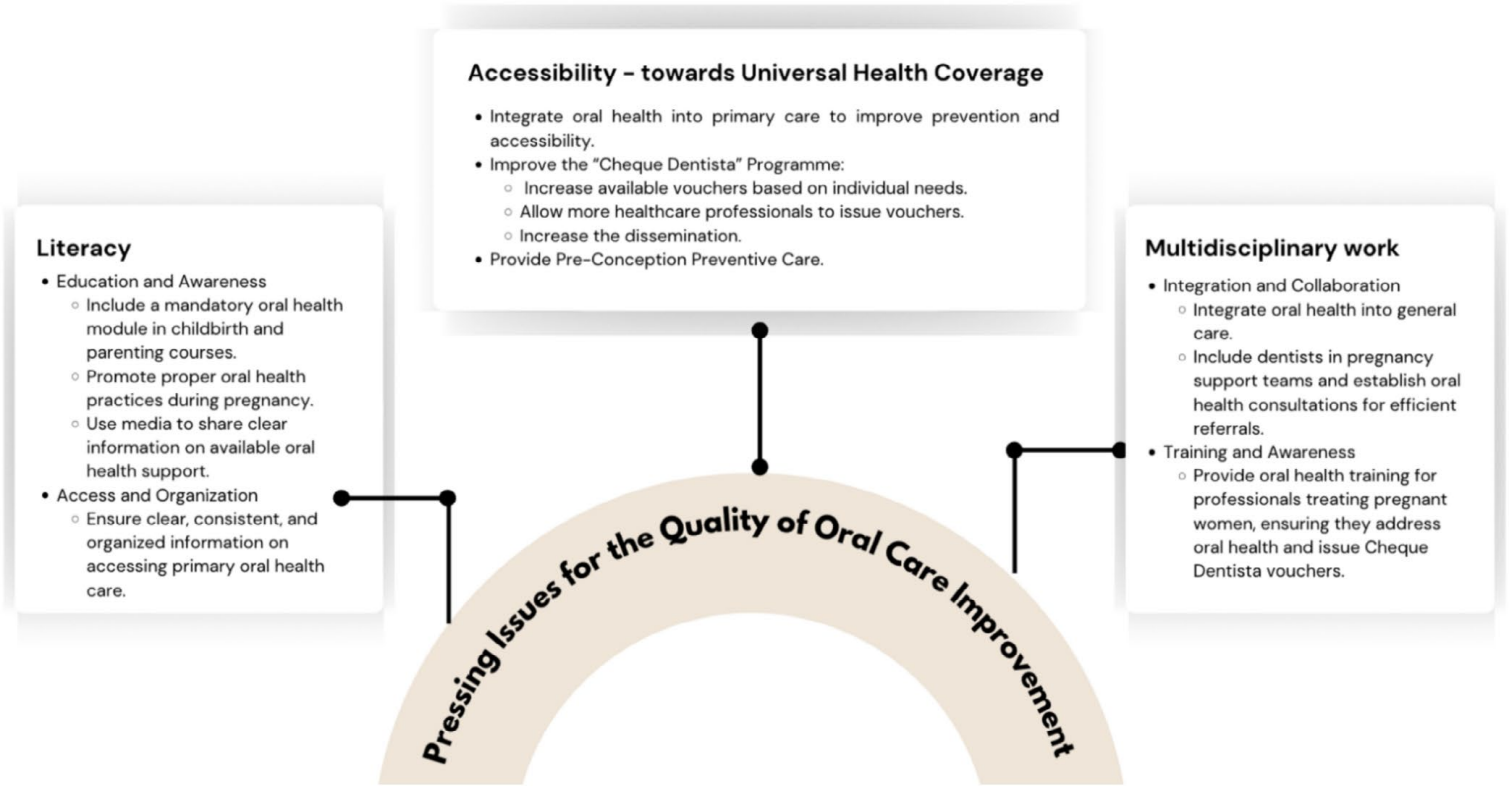
Three major categories of pressing issues. [Color figure can be viewed at wileyonlinelibrary.com]

This study applied the NGT to capture the perspectives of diverse stakeholders and identify pressing issues in maternal oral healthcare across three analytical levels: policy, community, and practice. The prioritisation process revealed both areas of divergence and convergence. At the policy level, priorities are centered on improving access through coverage expansion, structured diagnostic appointments, and integration of oral health into general health pathways. At the community level, the emphasis was on prevention, encompassing family planning consultations, training for health professionals, and direct patient engagement. At the practice level, stakeholders emphasised the importance of literacy and teamwork, including the integration of oral health education into childbirth preparation and the enhancement of multidisciplinary care teams. Across all three levels, three primary categories emerge as critical: literacy, accessibility, and multidisciplinary collaboration (Figure 4).

The integration of oral healthcare within primary care settings is considered essential for enhancing access to and the quality of oral health services. It has been suggested that appointments for oral health diagnostics should be systematically incorporated. This initiative aligns with the World Health Organization's (WHO) Action 63, which advocates for integrating oral health services as part of Universal Health Coverage (UHC), which includes expanding coverage through on‐demand care within primary care facilities and utilizing an essential oral health care package [5]. Despite its importance, dental care is often overlooked in health policy and treated as a discretionary, privately financed service [6]. This separation from primary care has led to significant affordability challenges, particularly for vulnerable populations, thereby limiting access and the potential for improving overall health and well‐being [6]. In many countries, unmet needs for dental care are reported more frequently than for medical care [20]. This disparity stems from the exclusion or partial inclusion in public health schemes [21]. For instance, in 2023, over 8% of individuals in Greece, Latvia, Portugal, and Denmark reported unmet dental care needs, primarily due to systemic barriers, with financial constraints being a significant factor [21]. This situation highlights the inadequate coverage of dental services for most citizens provided by the Portuguese National Health Service (NHS). Thus, there is an urgent need to reform the healthcare system to achieve UHC, which must encompass oral health services. In 2008, Portugal's NHS implemented a voucher system known as the *Cheque‐Dentista* Program, which enables eligible individuals, including pregnant women, to receive regular check‐ups and preventive treatments at no cost [8]. However, feedback from our participants indicates a need to increase the number of vouchers allocated for pregnant women within the *Cheque‐Dentista* Program and to extend this initiative to include postpartum care. To improve program utilization, policymakers could simplify referral pathways by implementing automatic voucher assignment, expanding eligibility to cover the preconception and postpartum periods, and reallocating resources, as unused vouchers could be assigned to women in need. Furthermore, participants considered a pressing need for enhanced communication regarding this program, as one of the principal reasons cited for its limited use is the lack of awareness among pregnant women, compounded by a general underappreciation of the importance of oral healthcare. This results in the subsequent challenge of insufficient literacy in oral health [22].

The participants considered it imperative to enhance the literacy of pregnant women and clarify the significance of oral health during preconception appointments. This initiative aligns with WHO Action 59, which focuses on improving the quality of oral healthcare through ongoing education [5]. In Malaysia, a component of the antenatal care visit protocol established within public healthcare facilities includes a dental screening conducted at the dental clinic situated within the health center [6]. As identified in a systematic review, barriers to accessing oral healthcare stem largely from inadequate literacy levels [3], compounded by a lack of knowledge about the severity and complications of oral health diseases [23]. This makes it essential to address misconceptions and emphasize the importance of oral health during prenatal care (e.g., in family planning consultations, childbirth preparation training…). Moreover, as stated in the WHO Action 29, there is a need to review and scale up mid‐stream promotion and prevention measures, including empowering women as change agents in families and communities [5, 24]. Practical interventions could include integrating oral health modules into childbirth and parenting classes, incorporating oral health counseling into family planning consultations, developing an easy‐to‐access online platform with oral health information, and distributing pamphlets with relevant information during maternal appointments. However, to address this, healthcare professionals should be able to address these topics [25].

It's also mentioned in the literature that prenatal care providers, such as midwives, gynecologists, and general practitioners, have a unique potential to assess and improve maternal oral health [26, 27, 28]. The systematic review conducted by Anunciação et al. [25] reveals that obstetricians often lack essential knowledge in this domain. Empowering other healthcare professionals and incorporating dental practitioners into maternal care teams can enhance health outcomes by adopting a holistic approach and enabling the successful scale of preventive care [25, 29, 30]. Therefore, training curricula for healthcare professionals should be updated to include oral health modules. At the same time, structured referral pathways between maternity care and dental services should be institutionalized at the health centers. As stated by Agili and Khalaf [31], the engagement of oral and prenatal healthcare providers in evidence‐based oral health promotion practices, antenatal dental collaboration, and the completion of the referral loop significantly enhances pregnant women's access to and utilization of preventive and therapeutic dental services.

Analysis of stakeholder voting revealed that stakeholder priorities were influenced by their background. Recent mothers consistently rated immediate and visible services, such as diagnostic appointments and direct consultations, as most urgent, reflecting a user‐centered demand for timely and tangible care. Dentists and general practitioners emphasized structural changes, including the integration of oral health into pregnancy support teams and adherence to national guidelines, indicating professional concern for systemic consistency and quality assurance. Policymakers prioritized coverage expansion, particularly enhancing the Cheque‐Dentista Program, indicating a focus on financial accessibility and scalability. These findings suggest that successful implementation in Portugal requires a multipronged approach that balances immediate user benefits (to satisfy mothers' needs), systematic integration (to address professional concerns), and scalable policy reforms (to meet policymakers' requirements for sustainability). The convergence around literacy, accessibility, and collaboration themes provides a framework for coordinated action across these different implementation perspectives.

This paper presents an innovative and comprehensive consensus list highlighting pressing issues related to oral care quality during pregnancy. It offers previously unavailable insights, concentrating on two primary aspects: (i) pressing issues in oral care practices during pregnancy and (ii) inconsistencies observed at the practice, community, and policy levels. We organized online meetings to convene various stakeholders, enabling a unique analysis from multiple perspectives. Including these stakeholders is essential for tackling the challenges threatening the quality of oral healthcare, emphasizing the need for collaborative efforts among citizens/patients, healthcare providers, and policymakers. We were unable to include pregnant women as participants. However, our participants were recent mothers who had experienced the pregnancy period and were able to testify more accurately. NGT ensures structured and inclusive decision‐making through a systematic ranking and voting process [32]. This method objectively prioritizes key pressing issues, generating comprehensive, stakeholder‐driven insights that may lead to more focused and actionable outcomes [32].

Limitations should also be acknowledged. The small sample size and snowball sampling strategy may introduce selection bias, limiting the generalizability of the results. The absence of pregnant women restricts the direct applicability of the findings, although recent mothers provided relevant lived experiences. Conducting NGT online may have influenced group dynamics compared to in‐person sessions. Finally, the consensus represents a temporal snapshot that may evolve alongside health system and policy developments.

Improving maternal oral healthcare in Portugal requires coordinated action at three levels. At the policy level, reforms should update the Cheque‐Dentista program by expanding voucher issuance to frontline professionals and the SNS24 line, reducing administrative barriers, and adjusting voucher value and scope to match treatment costs and population needs. Unused vouchers should be reallocated to women in need, while oral health records must be integrated into a unified clinical platform, with a focus on investment in underserved areas. Mandatory oral health check‐ups and efficient referral pathways should also be embedded in prenatal care. At the community level, oral health literacy should be incorporated into family planning and childbirth preparation, with accessible, reliable information. At the practice level, dentists should be integrated into maternal care teams, fostering structured multidisciplinary collaboration. Future research should broaden stakeholder representation and update national oral health surveys to generate robust evidence for policy development.

## Conflicts of Interest

The authors declare no conflicts of interest.

## Data Availability

The data that support the findings of this study are available from the corresponding author upon reasonable request.

## References

[1] N. Jain , U. Dutt , I. Radenkov , and S. Jain , “WHO'S Global Oral Health Status Report 2022: Actions, Discussion and Implementation,” Oral Diseases 30 (2023): 73–79. 36680388 10.1111/odi.14516

[2] S. S. Jahan , E. Hoque Apu , Z. Z. Sultana , M. I. Islam , and N. Siddika , “Oral Healthcare During Pregnancy: Its Importance and Challenges in Lower‐Middle‐Income Countries (LMICs),” International Journal of Environmental Research and Public Health 19, no. 17 (2022): 10681. 36078397 10.3390/ijerph191710681 PMC9518121

[3] L. Frey‐Furtado , M. Fonseca , P. Melo , S. Listl , and M. L. Pereira , “Oral Healthcare Access: Self‐Perceived Barriers Faced During Pregnancy—A Systematic Review,” BMC Public Health 25, no. 1 (2025): 1394. 40229781 10.1186/s12889-025-22593-8 PMC11995530

[4] Institute of Medicine Committee on Quality of Health Care in America , Crossing the Quality Chasm: A New Health System for the 21st Century (National Academies Press, 2001). 25057539

[5] WHO , Global Strategy and Action Plan on Oral Health 2023–2030 (World Health Organization, 2024), 104.

[6] World Economic Forum , The Economic Rationale for a Global Commitment to Invest in Oral Health (World Economic Forum, 2024).

[7] A. Sheiham and R. G. Watt , “The Common Risk Factor Approach: A Rational Basis for Promoting Oral Health,” Community Dentistry and Oral Epidemiology 28, no. 6 (2000): 399–406. 11106011 10.1034/j.1600-0528.2000.028006399.x

[8] J. Simões , G. F. Augusto , A. do Céu , et al., “Ten Years Since the 2008 Introduction of Dental Vouchers in the Portuguese NHS,” Health Policy 122, no. 8 (2018): 803–807. 30054096 10.1016/j.healthpol.2018.07.013

[9] Expresso , “68% Dos Cheques Dentista Emitidos em 2023 Foram Utilizados, um Valor Abaixo da Meta Estabelecida Pela DGS,” 2024, https://expresso.pt/sociedade/saude/2024‐03‐20‐68‐dos‐cheques‐dentista‐emitidos‐em‐2023‐foram‐utilizados‐um‐valor‐abaixo‐da‐meta‐estabelecida‐pela‐DGS‐fd12680f.

[10] NHS , “Get Help With Dental Costs,” (2025), https://www.nhs.uk/nhs‐services/dentists/get‐help‐with‐dental‐costs/.

[11] L. Frey‐Furtado , P. Melo , Á. Azevedo , S. Listl , and M. L. Pereira , “Knowledge and Attitudes of Dental Students Towards Treatment of Pregnant Women: A Cross‐Sectional Study,” European Journal of Dental Education (2025), 10.1111/eje.13103. PMC12834538 40259800

[12] A. Antunes , V. Rosete , and J. Fagulha , “Oral Health in Pregnancy,” Acta Médica Portuguesa 14, no. 4 (2001): 385–393. 11762179

[13] J. L. Esteves MP , C. Gomes , B. Cunha , A. Messias , and A. L. Costa , “Oral Health‐Related Knowledge and Practices Among a Cohort of Pregnant Portuguese Women,” Revista Portuguesa de Estomatologia, Medicina Dentária e Cirurgia Maxilofacial 62, no. 4 (2021): 229–236.

[14] D. Benatru Antunes CM , A. Figueiredo , and M. Seabra , “Medicina Dentária e Saúde Oral na Gestação—Estudo Piloto,” Revista Portuguesa de Estomatologia, Medicina Dentária e Cirurgia Maxilofacial 57 (2016): 1–61.

[15] N. Laurisz , M. Ćwiklicki , M. Żabiński , R. Canestrino , and P. Magliocca , “Co‐Creation in Health 4.0 as a New Solution for a New Era,” Healthcare 11, no. 3 (2023): 363. 36766938 10.3390/healthcare11030363 PMC9913923

[16] F. Khurshid , E. O'Connor , R. Thompson , and I. Hegazi , “Twelve Tips for Adopting the Virtual Nominal Group Technique (vNGT) in Medical Education Research,” MedEdPublish 13 (2023): 18. 37484833 10.12688/mep.19603.1 PMC10362375

[17] K. L. Matthews , M. Baird , and G. Duchesne , “Using Online Meeting Software to Facilitate Geographically Dispersed Focus Groups for Health Workforce Research,” Qualitative Health Research 28, no. 10 (2018): 1621–1628. 29911490 10.1177/1049732318782167

[18] T. Maguire , L. Garvey , J. Ryan , M. Olasoji , and G. Willets , “Using the Nominal Group Technique to Determine a Nursing Framework for a Forensic Mental Health Service: A Discussion Paper,” International Journal of Mental Health Nursing 31, no. 4 (2022): 1030–1038. 35591773 10.1111/inm.13023 PMC9321579

[19] P. Melo , L. Frey‐Furtado , D. Correia , et al., “Pressing Issues for Oral Care Quality Improvement: Findings From the EU DELIVER Project,” BMC Public Health 24, no. 1 (2024): 2173. 39134993 10.1186/s12889-024-19707-z PMC11318123

[20] OECD, European Observatory on Health Systems and Policies , Portugal: Country Health Profile 2023, State of Health in the EU (OECD Publishing, European Observatory on Health Systems and Policies, 2023).

[21] European Commission , Health at a Glance: Europe 2024: State of Health in the EU Cycle (OCED, 2024).

[22] C. A. Vamos , L. Merrell , T. A. Livingston , et al., ““I Didn't Know”: Pregnant Women's Oral Health Literacy Experiences and Future Intervention Preferences,” Women's Health Issues 29, no. 6 (2019): 522–528. 31235347 10.1016/j.whi.2019.05.005

[23] N. Phoosuwan , P. Bunnatee , and P. C. Lundberg , “Oral Health Knowledge, Literacy and Behavior of Pregnant Women: A Qualitative Study in a Northeastern Province of Thailand,” BMC Oral Health 24, no. 1 (2024): 653. 38834970 10.1186/s12903-024-04414-3 PMC11149361

[24] Economist Impact , Time to Put Your Money Where Your Mouth Is ‐ Addressing Inequalities in Oral Health (Economist Impact, 2024). 10.1038/s41415-024-7554-x 38942860

[25] B. H. Anunciação , M. J. Azevedo , and M. L. Pereira , “Knowledge, Attitudes, and Practices of Prenatal Care Practitioners Regarding Oral Health in Pregnancy—A Systematic Review,” International Journal of Gynecology & Obstetrics 162, no. 2 (2023): 449–461. 36710529 10.1002/ijgo.14703

[26] A. George , M. Johnson , A. Blinkhorn , S. Ellis , S. Bhole , and S. Ajwani , “Promoting Oral Health During Pregnancy: Current Evidence and Implications for Australian Midwives,” Journal of Clinical Nursing 19, no. 23–24 (2010): 3324–3333. 20955483 10.1111/j.1365-2702.2010.03426.x

[27] A. Kobylińska , N. Sochacki‐Wójcicka , N. Dacyna , et al., “The Role of the Gynaecologist in the Promotion and Maintenance of Oral Health During Pregnancy,” Ginekologia Polska 89, no. 3 (2018): 120–124. 29664546 10.5603/GP.a2018.0021

[28] R. Al‐Habashneh , S. H. Aljundi , and H. A. Alwaeli , “Survey of Medical Doctors' Attitudes and Knowledge of the Association Between Oral Health and Pregnancy Outcomes,” International Journal of Dental Hygiene 6, no. 3 (2008): 214–220. 18768026 10.1111/j.1601-5037.2008.00320.x

[29] S. Patil , R. Thakur , M. Kakanur , S. T. Paul , and P. Gadicherla , “Oral Health Coalition: Knowledge, Attitude, Practice Behaviours Among Gynaecologists and Dental Practitioners,” Journal of International Oral Health 5, no. 1 (2013): 8–15. PMC3768076 24155572

[30] The Economist Group , Time to Put Your Money Where Your Mouth Is: Addressing Inequalities in Oral Health (Economist Group, 2024).

[31] D. E. Al Agili and Z. I. Khalaf , “The Role of Oral and Prenatal Healthcare Providers in the Promotion of Oral Health for Pregnant Women,” BMC Pregnancy and Childbirth 23, no. 1 (2023): 313. 37138232 10.1186/s12884-023-05654-x PMC10157922

[32] A. H. Van de Ven and A. L. Delbecq , “The Nominal Group as a Research Instrument for Exploratory Health Studies,” American Journal of Public Health 62, no. 3 (1972): 337–342. 5011164 10.2105/ajph.62.3.337 PMC1530096

